# A Lived Experience of Mindfulness-Based Mentalizing Training Program for Parents (MBMP). A Phenomenological Study

**DOI:** 10.1177/13591045261422590

**Published:** 2026-02-23

**Authors:** Timo Teräsahjo, Sanna Herkama, Christina Salmivalli, Oskari Lahtinen

**Affiliations:** 1 INVEST Research Flagship Centre, University of Turku, Turku, Finland; 2 Research Centre for Child Psychiatry, University of Turku, Turku, Finland; 3 Department of psychology and speech-language pathology, University of Turku, Turku, Finland

**Keywords:** parental mentalizing, mindfulness, intervention, qualitative, phenomenological study

## Abstract

Mentalizing, or the ability to understand the mental states underlying oneself and others’ overt behaviors, is crucial in parenting due to its association with favorable child development outcomes. This study introduces a new tool, the Mindfulness-Based Mentalizing Training Program for Parents (MBMP), and investigates its potential to enhance parental mentalizing through qualitative analysis. Six parents of young children were interviewed to explore their lived experiences with MBMP using descriptive phenomenological analysis. The individual experiences were categorized into four main content areas: (1) The Experience of Mind Check: Observing internal states; (2) Power of Positivity: Recalling positive interactions; (3) Living Through Difficulties: Reflecting on challenging interactions; (4) Telling and Retelling: Describing the child from the parent’s perspective. The study discusses the key experiences stimulated by MBMP and its potential for enhancing parental mentalizing.

## Plain Language Summary

A Short, Audio-Guided Tool to Support Parent–Child Connection: Parents’ Views. Mentalizing means noticing the feelings and thoughts behind behavior—your own and your child’s. This study tried a new, short, audio-guided program for parents (Mindfulness-based Mentalizing Training Program for Parents, MBMP) that aims to strengthen this skill. Six parents of young children used MBMP at home and then took part in in-depth interviews. Parents described four kinds of experiences: (1) Mind Check: noticing their own body signals, emotions, and thoughts; (2) Power of Positivity: recalling everyday moments that felt good for both parent and child; (3) Living Through Difficulties: thinking about tough interactions and what each of them might have felt; (4) Telling and Retelling: describing the child at the beginning and again at the end—did anything change? Many parents said they could describe their child in more detail and with more nuance at the end. The exercises sometimes stirred positive feelings linked to good moments with the child. However, some parents also experienced strong negative feelings during stressful times. Overall, MBMP may help parents respond more thoughtfully in daily life. Because this was a small, qualitative study, we can’t tell how well it works for all families—larger studies are needed.

## Introduction

Mentalization, or mentalizing, refers to the ability to understand the mental states—both of oneself and others—that underlie overt behavior (Bateman & Fonagy, 2016). It involves interpreting the feelings, thoughts, beliefs, and wishes that explain what people do (Fonagy & Bateman, 2019). The capacity for mentalizing is essential for mental health and is commonly associated with recovery across a range of psychotherapies and interventions (Luyten et al., 2020). Increasing mentalizing in individuals, families, and society as a whole has the potential to enhance well-being and reduce conflicts (Campbell & Allison, 2022). We suggest that a cost-effective approach is to use an already widespread form of mental training—mindfulness meditation—and modify it to help to strengthen individuals’ capacity for mentalizing. In the current study, we analyze parents’ experiences while using a new tool, the Mindfulness-Based Mentalizing Training Program for Parents (MBMP), designed to stimulate parental mentalizing.

According to the two-component model of mindfulness proposed by Bishop et al. (2004), mindfulness involves (1) self-regulation of attention and (2) adopting an orientation characterized by curiosity, openness, and acceptance. A distinction can be made between *mindful awareness* and the *mindfulness practice* (Shapiro et al., 2024). Mindful awareness is a non-conceptual, receptive awareness of the present moment (Brown & Ryan, 2003; Shapiro et al., 2024), whereas mindfulness practice refers to the intentional cultivation of this state through metacognitive training that enhances attention regulation and emotional openness (Bishop et al., 2004; Shapiro et al., 2023). Mindfulness practice has been shown to be effective in various formats, such as 8-week Mindfulness-Based Stress Reduction (MBSR) courses (de Vibe et al., 2017), online programs (Lahtinen & Salmivalli, 2020; Ma et al., 2018), and as an integrated component in psychotherapies (Beygi et al., 2023; Panos et al., 2014).

Beyond defining mindfulness, Bishop et al.’s model also provides a conceptual bridge for linking mindfulness practice to effective mentalizing processes. The first component, sustained attention to present moment experience, maintains a receptive wakefulness that supports attunement to others’ signals and changes in mental states. It enables the detection of subtle shifts in one’s own and others’ psychological processes, and by keeping the flow of experience available to awareness, it enables more accurate reflection. This attentional stance also facilitates recognition of when a shift from implicit (automatic) to explicit (deliberate) mentalizing is needed—for instance, during emotional disruption or misunderstanding. The second component, an accepting and non-defensive orientation to experience, protects this attentional process from distortion. By fostering openness and reducing defensiveness, it supports mentalization-based emotion regulation (Török & Kéri, 2022) and allows fuller engagement with one’s own and others’ subjective experiences. Such openness resonates with Slade’s (2005) description of healthy mentalizing as a “non-defensive willingness to engage emotionally and make meaning of feelings and internal experiences without becoming overwhelmed or shutting down.” From this perspective, mindful awareness can be seen as supporting mature mentalizing, paralleling the *mentalizing stance* (Allen, 2013), characterized by a curious and not-knowing attitude toward mental states. Empirical studies consistently link mindfulness with mentalization across diverse contexts (Jalilvand & Ahmadi, 2023; Tomasino & Fabbro, 2016; Török & Kéri, 2022; Asgarizadeh & Ghanbari, 2024; Marszał & Górska, 2015).

In various interventions, mindfulness has already been integrated with mentalization in different ways. For example, Grienenberger et al. (2015) describe a model of early intervention and prevention that combines mentalization and mindfulness meditation through Mindful Parenting and Reflective Parenting groups, collectively referred to as Mindful and Reflective Parenting. Burke et al. (2020) developed a four-week intervention for university students that incorporates elements of mindfulness, mindful self-compassion, and mentalization-based interventions. Fan et al. (2024) explored the impact of combining Mindfulness Therapy with Mentalization-Based Family Therapy (MBFT) on suicidal ideation in adolescents with depressive disorders. Mentalizing Imagery Therapy (MI-Therapy) (Jain et al., 2022; Jain & Fonagy, 2020) offers mindfulness-based imagery training for psychotherapy clients and caregivers of patients with Alzheimer’s disease and related dementias. In psychotherapy, mindfulness and mentalization have been described as complementary qualities of the therapist’s stance that support reflective, emotionally attuned engagement (Allen, 2013; Wallin, 2007).

In the present study, we are particularly interested in strengthening mentalization in the context of parenting, i.e. fostering *parental mentalizing* - a concept that refers to the parental capacity to represent their child as a psychological agent and the parent’s proclivity to understand and interpret the child’s behavior in terms of mental states (Medrea & Benga, 2021). Subsequent research has consistently shown that parental mentalizing plays a crucial role in fostering children’s socioemotional and cognitive development (Meins et al., 2002; Moreira et al., 2024; Rosso & Airaldi, 2016; Shai & Belsky, 2017; Shai et al., 2022; Zeegers et al., 2017). Yet many established parenting programs focus primarily on behavior management and psychoeducation, sometimes overlooking parents’ capacity to reflect on their own and their child’s inner experiences. Developing interventions that explicitly foster parental mentalizing may therefore fill an important gap by complementing existing programs and targeting the deeper relational processes that underlie sensitive caregiving and secure attachment (Zeegers et al., 2017). The present study introduces such an approach in the form of MBMP, a brief and low-cost audio-based intervention that can be flexibly integrated alongside existing parenting programs.

## The Present Study

In the present study, we explore in depth the lived experiences of parents utilizing the MBMP. We describe the experiences stimulated by the exercises and, furthermore, focus on the potential of MBMP to enhance parental mentalization.

## Materials and Methods

### Intervention: Mindfulness-based Mentalizing Training Program for Parents (MBMP)

The MBMP is a brief audio intervention developed by the first author, designed to stimulate self-mentalizing (understanding one’s own mind) and child-mentalizing (understanding the child’s mind). It consists of ten 10–13-min sessions, each including two core exercises.

*The* Mind *Check* invites parents to notice bodily sensations, emotions, and thoughts in the present moment. This is meant to stimulate mindful awareness. *The Recalled Parent–Child Interaction* guides parents to: (1) remember an interaction with their child and imagine it vividly; (2) reflect on their own thoughts, feelings, and sensations in that situation; (3) imagine more precisely what the child looked like; (4) guess what kind of thoughts, feelings, and sensations their child may have experienced; and (5) notice what this imagery evokes (thoughts, feelings, sensations) in them now.

The Recalled Parent–Child Interaction is informed by mentalization theory and therapy (Bateman & Fonagy, 2016, 2019). Its structure mirrors the mentalizing process by first establishing a clear situational understanding, then turning to mental states, integrating implicit and explicit experience, balancing external and internal perspectives, and maintaining awareness of the distinction between minds and the inherent opacity of mental states.

In the first and final sessions, parents are asked to bring their child vividly to mind and describe their qualities, interests, and frustrations. This task is intended to enrich the child’s representation in the parent’s mind. Parents with more than one child could choose to focus on one or several children. The program also includes other elements. Its overall structure is presented in Table 1. A detailed intervention manual (currently in Finnish; English translation available upon request) can be obtained from the first author.

**Table 1.** Mindfulness-based Mentalizing Training Program for Parents (MBMP)

Training sessions	Content of exercise
Training session 1.	Introduction: Mind check + describe your child
Training session 2.	Mind check + positive interaction situation
Training session 3.	Mind check + positive or neutral interaction situation
Training session 4.	Mind check + positive or neutral interaction situation
Training session 5.	Mind check + being with difficult emotion
Training session 6.	Mind check + challenging interaction situation
Training session 7.	Mind check + optional interaction situation
Training session 8.	Mind check + optional interaction situation
Training session 9.	Mind check + optional interactional situation + compassion
Training session 10.	Mind check + describe your child

### Target Group

The MBMP is primarily targeted at parents of children aged approximately two to six years. MBMP may also be suitable for parents of children of other ages, but it is primarily designed with a focus on parent-child interaction during the toddler years.

### Participants

The interviewees were found through email lists and social media in Finland. Informed consent was obtained from all participants. Participants expressed their willingness to take part in the study after being introduced to the MBMP and its aims through a brief written description of the program. The participants had completed at least a secondary education. Basic information about the interviewees and their child (ren) is summarized in Table 2.

**Table 2.** Background Information of Interviewees and Their Children

Interviewees	Children
H, mother	Girl, 2.5 years
K, mother	Boys 4 and 6 years
N, mother	Girl, under 1 year
S, mother	Boys 2 and 6 years, girl 4 years
L, father	Boy 2 years, girl 5 years
J, father	Boys 6 and 7 years

*Note.* The initials are pseudonyms unrelated to the names of the interviewees.

### Procedure

The procedure followed the ethical standards of the University of Turku Ethics Committee for Human Sciences, Finnish National Board of Research Integrity (Finnish National Board on Research Integrity TENK, 2019), and the Finnish Personal Data Act (523/1999). The data collection procedure was consistent with the Finnish Human Subjects Protection regulations. All procedures performed in studies involving human participants were in accordance with the ethical standards of the institutional and/or national research committee and with the 1964 Helsinki Declaration and its later amendments.

### Conducting Interviews

Semi-structured interviews were conducted by the first author during the summer and fall of 2023, a few days to a couple of weeks after participants completed MBMP. The interviews were recorded via Zoom, except for one on-site interview. The purpose of the interviews was to obtain a detailed description of participants’ thoughts, feelings, bodily sensations related to using the program, and its significance in everyday life, particularly concerning interactions with their child. The interviews ranged from 40 min to 1 hour and 20 min in length. The interview data were transcribed verbatim by the first author, with each transcript spanning 12–15 pages (Times New Roman, 12 pt). The interviews were conducted in Finnish. Quotes presented in the results were translated into English by the first author. To ensure accuracy, a back-translation was carried out by an English language expert, which led to minor refinements in wording.

### Analysis: The Descriptive Phenomenological Psychological Method

The descriptive phenomenological psychological method (Giorgi, 1985; Giorgi et al., 2017) was chosen as the analysis method. This qualitative research approach is particularly suitable for investigating phenomena about which little is known, making it ideal for examining a novel training method with no existing data on user experience. The analysis was conducted by the first author and involved the following steps:

(1) *Reading and reflecting for a sense of whole.* The transcribed interviews were read with the audio through several times in an effort to form a general picture of the interviewed parent’s experience as a whole.

(2) *Assuming the attitude of scientific phenomenological reduction.* Special emphasis was given to *phenomenological reduction* referring to an effort to exclude theoretical underpinnings and to interpret the text as such, without the inference of prior knowledge and also to ensure that the researchers’ own personal experiences did not overshadow the experience of the interviewee. Attempts were made to cultivate a “not knowing” attitude in mind.

(3) *Division of transcript* *into meaning units focusing on phenomenon being studied.* The text was broken down to manageable *meaning units.* The meaning units were identified by reading the transcript more slowly, delineating each time that a transition in meaning in the parent’s experience was understood and interpreted the way it was expressed by the participant.

(4) *Transforming the everyday* *expressions into psychological language with emphasis on the phenomenon being investigated.* In this phase the original expressions of the participants, divided earlier in meaning units, were assigned psychological meanings so that the expressions utilized by the participants can be more directly apprehended (Giorgi, 1985). Example of transformative reflections are shown in Table 3.

**Table 3.** Example of Transformative Reflection

Original expression constituting a meaning unit	Transformation to psychological language
1. “Well, my child is timid or something like that. Maybe a bit slow to warm up and a fearful type ---though it affects all kind of things if one is a little bit slow to warm up, then maybe that kind of tenderness came to mind.”	1. While doing an exercise where H was asked to describe her own child in her mind, the child’s timidity, fearfulness, and tendency to warm up slowly came to H’s mind because these characteristics, according to H, have a significant impact on the child’s life.

(5) *Synthesis of the transformed* *meaning units into psychological structure of experience.* The transformations were reflected upon again, and texts concerning the same meaning were merged. Then, a new transformation was written, summarizing the respective meaning content, which took into account all transformations related to the topic. Finally, all summaries were integrated into a cohesive psychological structure that represents the individual’s overall experience. The resulting psychological structures, approximately one A4 page in length, were then validated by the interviewees, who were given the opportunity to refine the researcher’s compilation.

(6) *Categorizing individual experiences into content categories.* All validated psychological structures were categorized according to their relevance to different components of the MBMP. The aim was to clarify how each part of the MBMP was experienced and to facilitate a more comprehensive evaluation of the intervention.

## Results

The individual experiences of H, K, N, S, J, and L are presented through four content categories. Key experiences in individual structures have been compiled in Table 4.

**Table 4.** Content Categories and Key Experiences

Content category	Key experiences in individual level structures
The experience of mind check	Strong or relatively strong negative experience: emergence of suppressed emotions or bodily sensations (H, K, N)
	Increased awareness of internal mental state or body awareness (H, K, N, J, S, L)
	Sense of relief (H, N, S)
	Sense of progress in the exercise (H)
	Wandering of thoughts (S, J, K)
	Drowsy, sleepy feeling (J)
Recalling positive interaction situations	Positive bodily experiences (H, K, N, S, L, J)
	Positive feeling (joy, love etc.) (H, K, N, S, L, J)
	Positive impact on observation of/interaction with a child (H, N, S, J)
	Sense of accomplishment (N)
	Change in observing the child: Seeing positive aspects or recognizing the diversity of their personality (S, N)
Recalling challenging interaction situations	Emotionally strong or relatively intense experience, negative feelings and bodily experiences (H, N, S)
	Neutral or relatively neutral experience (L, J)
	Empathizing with the child’s perspective (H, K, N, S, L, J)
	Reflecting on one’s own mental state and the reasons for behavior (H, K, N, S, L, J)
The experience of describing the child in the mind of the parent	The description at the end of the program more subtle and profound (H, N, S, L, J)

### The Experience of Mind Check: Observing Internal States (Content Category 1)

The Mind Check exercise was perceived as a tool for revealing one’s own state of mind. The intensity and significance of the experience ranged from neutral to strong emotional reactions that stimulated deeper processing. The latter was particularly evident among parents who encountered the exercise program during a stressful life situation.

H, who was dealing with the stress of her spouse’s illness, found that the Mind Check exercise allowed her to confront troubling thoughts and emotions. The experience was accompanied by distressing bodily sensations. As she put it, “just like in my shoulders and somehow maybe in my chest, like a really strong distress.” K, burdened by parenting a child with special needs, also experienced the Mind Check exercise as revealing her own stress, which initially felt unpleasant. N, who encountered the exercise program during a highly stressful period, experienced even stronger emotions. The exercise brought suppressed feelings and thoughts to the surface, making it difficult for her to concentrate on subsequent exercises and engage with her child immediately after the exercise. As the program progressed and her stress levels slightly decreased, the anxiety stimulated by the Mind Check exercises also diminished.

Nevertheless, the experiences with Mind Check remained positive. H felt that, through the Mind Check, she was able to let go of thoughts circling in her mind by allowing the associated emotions to come into her awareness. As the training program progressed, strong emotions decreased, and the awareness of her internal state brought relief, along with a sense of improved focus and progress in the exercises. K, who initially perceived Mind Check as threatening, experienced a reduction in avoiding her inner states as the program advanced. By the end of the program, focusing on personal feelings and bodily sensations no longer seemed as threatening, and the exercises became more enjoyable. Despite the difficulty of confronting heavy thoughts and emotions, N perceived Mind Check as a “purifying” and “relieving” experience, finding it helpful in processing her stressful life situation.

A relieving experience related to processing life situations also emerged from S, who, however, did not describe the Mind Check as an emotionally heavy or intense experience. For her, the Mind Check exercise was positive and calming, enhancing body awareness. This effect was, however, associated with experiences similar to those previously mentioned, such as opening up and recognizing the current state of being. The positive significance was tied to an experience she described as a sense of relief, which was linked to seeing the reasons behind the current state of mind.

However, not all parents experienced a particular sense of relief or processing of their mental states from the Mind Check exercise. For J, the Mind Check exercises were neutral experiences: they were relaxing, reduced arousal, and induced what J described as “starting to make me a little tired” along with “thoughts are starting to wander.” During the exercise, he might have become aware of physical strain in his body. For L, who had a background in mindfulness meditation, the Mind Check exercises resembled his regular meditation practice, where he becomes aware of the content of his mind, including thoughts, feelings, and sensations, as they manifest. He did not perceive any particular heaviness associated with the exercises. Instead, the exercise appeared to manifest as a neutral awareness of his internal state.

### Power of Positivity: Recalling Positive Interactions (Content Category 2)

For parents, recalling positive interactions with the child felt good, and some parents experienced that it had implications for interacting with their child as well. All parents described both the mental states of their children and themselves. However, variation in experiences was apparent here as well.

H initially felt quite restless during the early stages of the program, and at that point, she found it confusing that she couldn’t remember what her child looked like when happy. She struggled to identify what situations might make a child happy and had trouble selecting specific situations to recall. As the program progressed, H experienced relief and relaxation, with less pressure in making choices. She decided to recall ordinary, everyday situations with her child, noticing that reflecting on positive moments helped her become more aware of similar instances in their daily life. K found it easier to reflect on positive interaction situations and think about her child in general than to focus on herself, partly because she had not had the opportunity to do so in her daily life. K noticed an improvement in her focus when the exercise was directed towards the child. She could recall various situations effortlessly from the beginning and reflected on her own mental states without strong anxiety. K found that thinking about positive interactions and aspects of her child made her feel good, even affecting her breathing by deepening it. She experienced feelings of pride and joy, for example. N felt that recalling her child’s joy and laughter evoked a strong sense of positivity. Thinking about positive situations often gave her “a kind of successful and sort of warm feeling.” For N, the successful feeling meant ease in the exercise and a sense of focus, characterized by “like an easy experience for me, or somehow that it kind of flowed.” Immediately after a successful exercise, N felt a closer connection with her child, and this positive feeling occasionally translated into her interactions with the child. After the exercise, N felt more conscious during interactions with her child, noticing positive aspects of the situation and the child.

S found recalling positive moments to be particularly meaningful and felt that these positive situations influenced her interactions with her child. She observed that reflecting on positive situations “brought out different personality shades” in her children and helped her respond more thoughtfully to the child’s impatience based on the child’s needs. S felt that recalling positive interactions was valuable because it mitigated the impact of negative experiences, and she eagerly chose positive interaction situations whenever given the opportunity in the training program. J also focused on positive situations when given the choice, but for him, the selection process was “kind of subconscious,” driven by positive mental images such as “the little guy’s laughing face” that came to mind during decision-making. L, on the other hand, found recalling positive situations to be a pleasant and “holistic, like warm feeling”, but he did not prioritize this aspect. He felt that he was more focused on handling challenging situations during the training program.

### Living Through Difficulties: Reflecting on Challenging Interactions (Content Category 3)

Thinking about challenging situations felt meaningful and constructive for the parents, although some also found this exercise burdensome. In this respect as well, the experiences of parents varied.

When recalling difficult situations, H experienced the same level of stress and a desire to escape from the situation that she had felt during the actual events. Additionally, she felt shame for losing control and reflected on her own behavior, wondering “whether it could have been done differently” in order to avoid being provoked by the child’s anger. Initially, H found it challenging to consider situations from the child’s perspective, but this became easier as the training program progressed. She began to notice similarities between herself and the child, which facilitated a better understanding of the child’s perspective in challenging situations. Reflecting on challenging interactions was not particularly distressing for K, although she recalled feelings of anger and “clenched teeth” from the situation. K felt that she had already processed the event and accepted such occurrences as part of family life. She generally found thinking about different interaction situations to be beneficial. Imagining the situation from the child’s perspective, especially from the perspective of a child with special needs, felt particularly meaningful to K. However, K noted that it had become a habit for her to process challenging situations with the child and attempt to verbalize the child’s emotions.

N also found the contemplation of challenging situations necessary, despite it being “an uncomfortable” exercise for her. Reflecting on these situations made N feel guilt for her own frustration. Considering the child’s perspective, especially regarding the child’s feelings and needs during night awakenings, intensified her sense of guilt. N also experienced anger directed towards the practice itself, which resurfaced negative emotions. After the exercise, N felt that the guilt and discomfort from reflecting on the situation subsided. She was able to contextualize and understand her own anger and actions through reflection. N found that thinking about another challenging situation became easier and less distressing over time. She noted that the structure of the exercise became familiar, allowing her to better orient herself to the task. N believed that reflecting on challenging situations provided a useful model for understanding both the situation and the child’s experience during those moments. For instance, she noticed that she automatically thought about the child’s experience in a manner aligned with the program’s model after challenging situations. Despite the difficulties, N viewed the contemplation of challenging situations as constructive, even acknowledging their emotional weight.

S felt that imagining the challenging situation evoked emotions and made her feel “tired.” During the exercises, she was able to clearly visualize interactive situations in her mind and experience the associated emotions. However, her emotional state did not escalate excessively. Since the situation was not actually occurring, it allowed her to reflect on both her own and the child’s experiences. Considering the child’s perspective and reflecting on her own feelings, especially in the absence of the child, helped to calm the emotions that arose, preventing lingering anxiety related to the situation. S perceived the reflection on challenging situations as positive and “relieving.”

J found recalling the situation to be primarily associated with a “neutral feeling”. The negative emotions associated with the situation did not resurface strongly in his mind. He noted that, in his recent history, he had not encountered situations where he experienced strong frustration or intense negative emotions related to his children. L, on the other hand, found it relatively easy to recall and identify challenging situations and his emotions in them. Considering the child’s perspective during the exercise was also quite effortless for him. L was able to empathize with the child’s mental state and understand its impact on the child’s behavior. A single exclamation from the child provided L with valuable insight into the child’s experience. L believes it is important to recognize his own emotions to avoid getting stuck in them in various situations. In the two challenging situations he recalled, the difficulty arose from the child’s resistance or undesired behavior and his own reactions. L has observed that being aware of his emotions, even if he cannot fully control or change them in the moment, leads him to revisit the situation with his children and apologize for his reactions when necessary.

### Telling and Retelling: Describing the Child from the Parent’s Perspective (Content Category 4)

Most of the parents felt that the description of the child (the exercise where a parent was asked to describe their child in their mind) at the end of the program differed from the description at the start of the program. They felt that the description at the end of the program was more subtle and profound.

H, who initially described her child as shy, fearful, and having a tendency to “warm up slowly,” felt that her perspective had changed by the end of the program. Although the child’s shyness remained the same, H now considered it more from the child’s perspective. She reflected on how separation from the mother could be “terribly stressful” for a sensitive child and how closeness could be reassuring. In this context, H found the exercise recalling memories of oneself as a child (compassion exercise, practice session 9) particularly important. It evoked for her the feeling of fear experienced as a child and the desire to be close to her mother. This led to a stronger sense of empathy towards her child’s experience. H also felt that recognizing the similarities between herself and her child helped her handle difficult mornings better. For K, describing her child in her mind was enjoyable at the beginning of the program. By the end of the training program, describing the child remained pleasant. However, K thought about her other child, and the descriptions were not comparable in the same way as with H.

N noticed a change in how she described her child from the beginning to the end of the training program. Initially, N found the exercise exciting and easy, but by the end of the program, she felt that the “child’s personality had grown.” This change was reflected in the nuanced differences in the adjectives she used, which N attributes to the development and growth she observed in her child. S initially found describing her child pleasant, though it also induced some fatigue due to the challenges in the daily life. By the end of the program, she felt that “even the difficult things seemed somehow clearer,” and she experienced more positive images of her child and feelings of affection. L also felt that by the end of the training program, he was able to describe his child in more depth and from a “next level” perspective than at the beginning. Similarly, J noticed that the exercises added “sensitivity” and “depth” to his description of his child, making it feel “warmer or more loving” compared to his initial descriptions.

“Now it kind of feels at least that […] I can more easily describe what my child is like and describe it more deeply […] if […] probably the purpose is to make some kind of review, like, whether this exercise has any impact, then I’d say that yes, it does have an impact. You can describe more deeply and […] put yourself in the child’s position and get at those nuances […] like, like, “oh, what a lively boy” and [he] told funny stories, so so you can then kind of get to the next level.” (L).

## Discussion

In the present study, we introduced and qualitatively investigated a new tool, the Mindfulness-Based Mentalizing Training Program (MBMP), to explore its potential for enhancing parental mentalizing. Notably, the program’s impact on stimulating mentalizing is evident in the parents’ experiences of describing their child. At both the beginning and end of the program, parents were guided to silently describe what their child is like.

Most parents reported that their descriptions were richer and more nuanced by the end of the program compared to the beginning, suggesting a perceived diversification of the child’s personality in the parents’ minds. Since the “richness of mentality” in describing the child is one measure of parental mentalization (McMahon & Bernier, 2017), we infer that this perceived change indicates a strengthening of child mentalizing, at least temporarily. This suggests that even brief mindfulness-based exercises, which involve recalling everyday situations and contemplating the child’s mind, can have a significant impact.

The descriptions also suggest that the exercises might have transfer effects to everyday life. Several parents noted that recalling positive interaction moments, in particular, had a positive impact on their perception of their child. In this context, the positive exercises may have acted as a form of *positive attachment security priming*, a phenomenon involving exposure to stimuli designed to activate feelings of love, comfort, and safety (Gillath & Karantzas, 2019).

Recalling challenging situations was generally perceived as meaningful and constructive. This suggests that the MBMP may have supported the repair of disruptions in mentalizing that often occur during challenging situations. However, recalling these situations was sometimes burdensome, which may indicate that the exercises occasionally triggered experiences that fell outside the optimal arousal range for mentalizing—where affective arousal is neither too low nor too high (Luyten et al., 2020).

The study also provided good insights into practicing in stressful life situations. The impact of stress was most evident in the Mind Check exercise, which evoked strong but ultimately “cleansing” or “relieving” experiences, suggesting further processing of stressful situations through mindfulness-related emotion regulation (Guendelman et al., 2017).

A stressful life situation did not appear to affect child-mentalizing as much as self-mentalizing. One reason for this difference may lie in the fact that the Mind Check exercise exposes the current life situation (and current stressors), and may even bring into awareness unmentalized contents of the mind (i.e., experiences that have not yet reached symbolized form). In contrast, recalling past situations with a child may render the imagined experiences more structured and imbued with temporal distance.

Our findings did not suggest a specific prioritization of self-over child-mentalizing. However, they may suggest a dynamic interplay between the two, where reflecting on one’s own experiences sometimes deepened the understanding of the child, and focusing on the child’s mind could, in turn, prompt reflection on oneself. This is consistent with earlier studies showing that self-mentalizing may, in certain contexts, be particularly important for quality of caregiving (Suchman et al., 2010), while other work highlights the value of child-focused mentalizing (McMahon & Bernier, 2017).

In summary, our study shows that the use of MBMP is experienced individually, depending on the parent’s life situation. Additionally, the results indicate that even brief exercises can stimulate mentalizing but may also evoke intense experiences, particularly in stressed parents. Such findings are consistent with evidence that mindfulness-based practices can sometimes elicit adverse experiences (Farias et al., 2020). Extending the listening period beyond the intended 10 days—as many parents did—appeared to be particularly helpful for processing the program’s content.

MBMP aligns with other emotion-focused interventions that aim to strengthen emotional understanding within families (Havighurst et al., 2020). Given its simple, low-cost format, it may be most naturally applied as a supportive complement to existing programs. For example, where deemed suitable, MBMP may support and deepen reflective work between sessions. MBMP may be particularly useful for parents who have a basic capacity for mentalizing but whose mentalizing capacity is challenged in the context of the child’s socioemotional difficulties, temperamental reactivity, or developmental issues (e.g., delayed language acquisition). Strengthening parental mentalizing in such contexts may help prevent the emergence of psychological difficulties in the child, as parental mentalizing has been shown to act as a protective factor for child mental health in most studies (Moreira et al., 2024).

Our study was limited by a small, motivated sample of parents with relatively intact mentalizing capacity, reducing generalizability. Broader and more diverse sampling, alongside rigorous designs such as randomized controlled trials and validated measures (e.g., the Parent Development Interview; Slade & Sleed, 2024), will be needed to evaluate MBMP’s effectiveness. As MBMP may evoke strong emotional responses and mindfulness-based practices can be challenging for vulnerable individuals (Kernberg, 2011), its application should be guided by clinical judgment.
